# Stability Indices to Deciphering the Genotype-by-Environment Interaction (GEI) Effect: An Applicable Review for Use in Plant Breeding Programs

**DOI:** 10.3390/plants11030414

**Published:** 2022-02-02

**Authors:** Alireza Pour-Aboughadareh, Marouf Khalili, Peter Poczai, Tiago Olivoto

**Affiliations:** 1 Seed and Plant Improvement Institute, Agricultural Research, Education and Extension Organization (AREEO), Karaj 31587-77871, Iran; 2 Department of Biotechnology and Plant Breeding, Payame Noor University, Tehran 14556-43183, Iran; 3 Botany Unit, Finnish Museum of Natural History, University of Helsinki, P.O. Box 7, FI-00014 Helsinki, Finland; 4 Department of Plant Sciences, Federal University of Santa Catarina, Florianópolis 88034-000 SC, Brazil; tiagoolivoto@gmail.com

**Keywords:** genotype-by-environment interaction (GEI), stability, GGE biplot, AMMI model, dynamic concept

## Abstract

Experiments measuring the interaction between genotypes and environments measure the spatial (e.g., locations) and temporal (e.g., years) separation and/or combination of these factors. The genotype-by-environment interaction (GEI) is very important in plant breeding programs. Over the past six decades, the propensity to model the GEI led to the development of several models and mathematical methods for deciphering GEI in multi-environmental trials (METs) called “stability analyses”. However, its size is hidden by the contribution of improved management in the yield increase, and for this reason comparisons of new with old varieties in a single experiment could reveal its real size. Due to the existence of inherent differences among proposed methods and analytical models, it is necessary for researchers that calculate stability indices, and ultimately select the superior genotypes, to dissect their usefulness. Thus, we have collected statistics, as well as models and their equations, to explore these methods further. This review introduces a complete set of parametric and non-parametric methods and models with a selection pattern based on each of them. Furthermore, we have aligned each method or statistic with a matched software, macro codes, and/or scripts.

## 1. Introduction

In multi-environment trials (METs) where a set of genotypes are tested in a set of environments (locations, years or combination of them), recommendation of genotypes to specific environments or delineation of mega-environments is the main plant breeding goal [1,2]. In such experiments, genotypes are usually tested in each environment in a randomized complete-block design with more than two replications. In these circumstances, METs facilitate the identification of genotypes that show a small variability or that are consistent across multiple locations. When a series of genotypes are examined in METs, in addition to the additive effect of genotype (G) and environment (E), a multiplicative effect arises from the interaction between these main factors, which is known as the genotype–environment interaction (GEI) effect. Indeed, the GEI is referred to the discordance of the genotype’s response in each environment. Hence, the GEI can be classified into two groups: (i) crossover or qualitative interaction and (ii) non-crossover of quantitative interaction. In the first definition, the differential response of genotypes to various environments is referred to as a crossover interaction when genotype ranks change from one environment to another. On the other hand, non-crossover interaction represents changes in the magnitude of genotype performance without change in rank order of genotypes across diverse environments. In this context, if the response of a genotype to environments is parallel to the mean response of all genotypes, it is identified as stable [3,4].

The propensity to model the GEI led to the development of a series of methods and approaches called “stability analyses”, the concepts of which precede even analysis of variance (ANOVA) [5]. The terms “performance stability” and “phenotypic stability” are usually used to refer to fluctuations in the phenotypic expression of crop performance while the genotypic composition of the genotypes remains stable [6]. Investigation of stability is important in any breeding program, where the GEI effect should be dissected. Leon [7] defined two concepts of stability based on the goal and on the characteristics under consideration, which are termed “static” and “dynamic” concepts of stability. In the static concept, a specific stable genotype has a performance that is unaffected by the environmental conditions. Furthermore, this concept is analogous to the biological concept of stability such that the yield performance of a stable genotype has an environmental variance near to zero [6]. The dynamic concept states that a stable genotype has no deviation from predictable response to environments. In other words, the performance of a stable genotype is accordance with the estimated level or the prediction for each environment. Thus, the genotypic response to environmental conditions is not equal for all genotypes. Becker [8] stated that this concept is analogous to the agronomic concept of stability, and most breeders prefer to apply it to select high yielding genotypes in their METs. However, it is worth noting that there is no absolute decision on classifying stability parameters based on dynamic and static concepts. For instance, in many studies, some stability parameters have a dynamic concept due to their correlation with yield performance. On the contrary, some studies reported a static concept for them. Hence, we believe that dynamic and static concepts depend on the nature of data and test environments and that classifying them in an absolute scale is not logical.

## 2. Importance of the GEI Effect

The importance of the GEI can be revealed from the relative contributions of the new varieties and followed by improved management to performance increases from direct comparisons of performances of them with old varieties in a single experiment [8]. According to available data, the genetic improvements have accounted for 50% of the total gains in performance per unit area for major crops over the past seven decades. Silvey [9] stated that GEI confounds partitioning of the contributions of improved cultivars and improved environment to the economic performance in different crop plants. The GEI has considerable effects on all stages of a breeding program and has several implications for the allocation of resources. For instance, a large GEI could mean the establishment of two target breeding environments (sites) in a region, instead of one, thus requiring increased input of resources [10]. The heritability of a quantitative trait such as grain yield plays a key role in determining genetic advances in the selection cycle. Indeed, heritability is the amount of phenotypic variation in a population that is attributable to individual genetic differences and is considered as a baseline of any breeding program [11]. As a component of the total phenotypic variance, the GEI has a negative effect on heritability. In other words, a large effect of the GEI results in smaller heritability; thus, progress from selection would be limited [8]. In METs, the best linear unbiased prediction (BLUP) is a useful model that will provide an ideal opportunity for the estimation of heritability across various environments [12,13]. Furthermore, this model provides additional information for the test of stability, adaptability, and simultaneous stability-adaptability in METs, which will be described in the following.

## 3. Deciphering GE Interaction Using Different Statistical Methods

**Figure 1:**
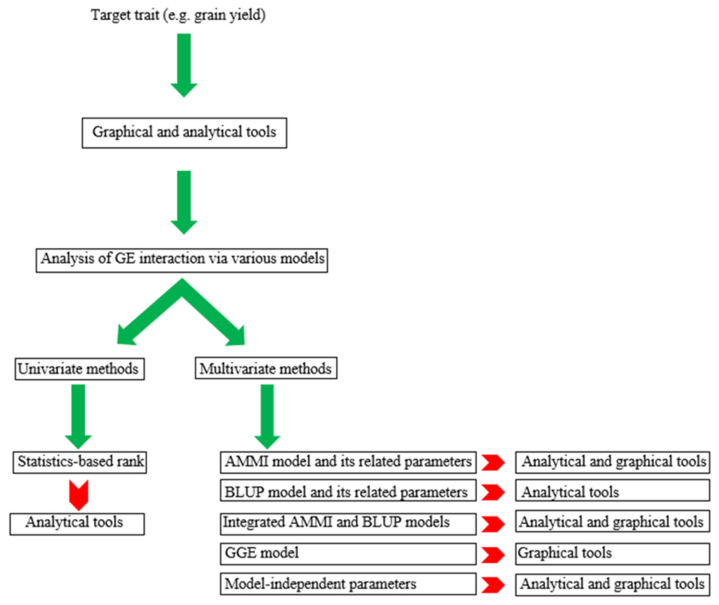
The guideline scheme of main groups of stability model.

Various statistical models and approaches have been proposed to analyze and interpret the GE interaction over environments and these can be divided into two major groups. The first group includes the parametric stability statistics that are further divided into two classes: (i) univariate and (ii) multivariate approaches (Figure 1). This group of stability statistics relies on distributional assumptions about environmental, genotypic, and their interaction effects. The second group is non-parametric stability statistics, which requires no primary assumptions. Non-parametric approaches are estimated based on the mean values of the response trait and ranking of genotypes. Indeed, this procedure results in the reduction of the bias caused by outliers, and no assumptions are needed about the distribution of observed values. Hence, the non-parametric statistics are easy to use and make it easy to decipher GEI, and additions or deletions of one or a few genotypes have little effect on the results [14]. In this way, if the breeder is only interested in the existence of rank-order differences across environments, the non-parametric statistics provide an excellent alternative to the parametric stability statistics currently used [15]. To comprehensively review the different stability statistics, we listed various methods and approaches. Table 1 shows the pattern of selection based on each calculated statistic and parameter.

**Table 1:** List of parametric and non-parametric stability statistics to analyze GEI effect in METs experiments.

Statistic	Symbol	Pattern of Selection	Type of Method	Year of Proposition	References
Environmental variance	S^2^	Minimum value	Parametric	1917	[16]
Mean variance component	θ	Minimum value	Parametric	1959	[17]
GE variance component	θ′	Maximum value	Parametric	1960	[18]
Wricke’s ecovalence	$W_{}^{2}$	Minimum value	Parametric	1962	[19]
Regression coefficient	b_i_	See Section 3.2.5	Parametric	1963	[20,21]
Deviation from regression	$S_{\mathrm{di}}^{2}$	Minimum value	Parametric	1966	[21]
Tai’s stability statistics	λ and α	Minimum value	Parametric	1971	[22]
Shukla’s stability variance	$\sigma_{}^{2}$	Minimum value	Parametric	1972	[23]
Pinthus’s coefficient of determination	R^2^	Maximum value	Parametric	1973	[24]
Coefficient of variance	CV	Minimum value	Parametric	1978	[25]
Nassar and Huhn’s and Huhn’s statistics	S^(1, 2, 3, 6)^	Minimum value	Non-parametric	1979	[26,27]
Superiority index	P	Maximum value	Parametric	1988	[28]
Kang’s rank-sum	KR	Minimum value	Non-parametric	1988	[29]
TOP-Fox	TOP	See Section 3.1.3	Non-parametric	1990	[30]
Yield stability index	YS	Maximum value	Parametric	1993	[31]
Averages of the squared eigenvector values	Ev	Minimum value	Parametric	1994	[32]
Thennarasu’s non-parametric statistics	NP^(1−4)^	Minimum value	Non-parametric	1995	[33]
Sums of the absolute value of the IPC scores	SIPC	Minimum value	Parametric	1997	[34]
Sum across environments of the GEI modeled by AMMI	AMGE	Minimum value	Parametric	1997	[34]
Distance of IPCAs point with origin in space	D	Minimum value	Parametric	1997–98	[35,36]
AMMI stability value	ASV	Minimum value	Parametric	2000	[37]
Stability measure based on fitted AMMI model	W_i(AMMI)_	Minimum value	Parametric	2002	[38]
AMMI Based Stability Parameter	ASTAB	Minimum value	Parametric	2005	[39]
Harmonic mean of genotypic values	HMGV	Minimum value	Parametric	2007	[40]
Relative performance of genotypic values	RPGV	Minimum value	Parametric	2007	[40]
Harmonic mean of RPGV	HMRPGV	Minimum value	Parametric	2007	[40]
Genotype stability index	GSI	Maximum value	Non-parametric	2008	[41]
Modified AMMI stability value	MASV	Minimum value	Parametric	2012	[42]
Absolute value of relative contribution of IPCAs	Za	Minimum value	Parametric	2012	[42]
Sum across environments of absolute value of GEI modeled by AMMI	AV_(AMGE)_	Minimum value	Parametric	2012	[42]
AMMI stability index	ASI	Minimum value	Parametric	2014	[43]
Modified AMMI stability index	MASI	Minimum value	Parametric	2018	[44]
Weighted average of absolute scores	WAASB	Minimum value	Parametric	2019	[45]

### 3.1. Non-Parametric Stability Statistics

#### 3.1.1. Huehn’s and Nassar and Huehn’s Statistics

In 1979, Huehn [26] proposed rank-based non-parametric stability statistics to rank genotypes in a MET. These proposed statistics have since been developed to incorporate the statistical properties and significance for the two first non-parametric methods (Z_1_ and Z_2_) suggested by Nassar and Huehn [27]. These statistics are (1) the mean of the absolute rank differences of a genotype over all tested environments (S^(1)^), (2) the variance among the ranks over all tested environments (S^(2)^), (3) the sum of the absolute deviations for each genotype relative to the mean of ranks (S^(3)^), and (4) the sum of squares of rank for each genotype relative to the mean of ranks (S^(6)^). To calculate these statistics, the mean yield data were transformed into ranks for each genotype and environment, and the genotypes are considered stable if their ranks are similar across environments. The lowest value for each of these statistics reveals high stability for a certain genotype. Additional details regarding mathematical relations are shown in the following formula:

$$S^{\left(1\right)}=\;2\;\sum_{j}^{n-1}\;\frac{\sum_{j^{\prime }=j+1}^{n}\left|r_{ij}-{r^{\prime }}_{ij}\right|}{\left[N(N-1)\right]}\;,\;S^{\left(2\right)}=\;\frac{\sum_{j=1}^{n}{\left(r_{ij}-{\bar{r}}_{i.}\right)}^{2}}{(N-1)}\;,\;\;\;\;S^{\left(3\right)}=\;\frac{\sum_{j=1}^{n}{\left(r_{ij}-{\bar{r}}_{i.}\right)}^{2}}{{\bar{r}}_{i.}}\;,\;\;\;S^{\left(6\right)}=\;\frac{\sum_{j=1}^{n}\left|r_{ij}-{\bar{r}}_{i.}\right|}{{\bar{r}}_{i.}}$$

where r_ij_ is the rank of the *i*th genotype in *j*th environment, r ®_ij_ is the mean rank across all environments for each genotype, and N is the number of test environments. Additionally, the significance tests, Z_1_ and Z_2_, for the S^(1)^ and S^(2)^ statistics were developed in a way that was similar to that suggested by Nassar and Huhn [27] as follows:

$$Z_{\left(m\right)}={\left[S_{i}^{\left(m\right)}-E(S_{i}^{\left(m\right)})\right]}^{2}/V(S_{i}^{\left(m\right)}),m=1,2$$

where E(Si^(m)^) is expectation of Si^(m)^, and V(Si^(m)^) is variance of Si^(m)^. Furthermore, these statistics are estimated according to the following equations:

$$\begin{array}{l} E(S_{}^{\left(1\right)})=(K^{2}-1)/3K,\;E(S_{}^{\left(2\right)})=(K^{2}-1)/12\\ V(S_{}^{\left(1\right)})=(K^{2}-1)[(K^{2}-4)(N+3)+30]/45K^{2}N(N-1)\\ V(S_{}^{\left(2\right)})=(K^{2}-1)[(2(K^{2}-4)(N-1)+5(K^{2}-4)]/360N(N-1) \end{array}$$

In the above equations, N and K are the number of environments and genotypes, respectively. Finally, the obtained Z values were tested by $\chi^{2}$ test.

#### 3.1.2. Kang’s Rank

This statistic was introduced by Kang [29] to select high-yielding and stable corn genotypes across various environments. This method, which is named Kang’s rank-sum (*RK*), uses both yield and Shukla’s stability variance (σ^2^_i_) as a selection index. This parameter gives a weight of one to both yield and stability statistics to identify high-yielding and stable genotypes. The genotype with the highest yield and lower σ^2^_i_ are assigned a rank of one. Then, the ranks of yield and stability variance are added for each genotype and the genotypes with the lowest rank-sum are the most desirable.

#### 3.1.3. TOP-Fox

As another non-parametric stability statistic, Fox et al. [30] suggested an ideal and simple parameter to superiority measure of general adaptability. This method is a stratified ranking approach that includes scoring the number of test environments in which each entry ranked in the LOW (bottom), MID (middle), and TOP (top) thirds of trial entries. The genotype that occurred mostly in the top third (high top value) was considered a widely adapted cultivar. The proportion of environments at which the genotype occurred in the each of these groups was determined to form the non-parametric measures of LOW, MID, and TOP, respectively. A high value of TOP (genotype that occurred mostly in the top third) was revealed to be a widely adopted genotype.

#### 3.1.4. Yield stability index (YS)

The yield stability (YS) statistic was introduced by Kang [31]. According to this method, a genotype with the highest mean yield is given the lowest rank (rank = 1). Similarly, a rank of 1 was assigned for the stability parameter with the lowest estimated value. Stability ratings were calculated as follows: −2, −4, and −8 for stability measures significant at *p* < 0.1, 0.05, and 0.01, respectively and 0 for the non-significant stability measure. The stability ratings of −2, −4, and −8 were selected because they changed genotype ranks from those based on the yield alone. Hence, this statistic would help breeders in selecting the genotypes with high and relatively stable yields across different environments as it integrates stability and yield performance of genotypes.

#### 3.1.5. Thennarasu’s Statistics

Since the rank of genotypes in the specific environments cannot be done according to the phenotypic values, the stability of test genotypes has to be estimated independently of the genotypic effect. To solve this challenge, a correction of ranking patterns of test genotypes and environments based on the corrected phenotypic values was developed. Four NP^(1–4)^ statistics are a set of alternative non-parametric stability statistics defined by Thennarasu [33]. Indeed, these parameters are based on the ranks of adjusted means of the genotypes in each environment.

The formulas to compute these statistics are shown below:

$$\begin{array}{l} NP^{\left(1\right)}=\frac{1}{N}\;\sum_{j=1}^{n}\left|r_{ij}^{*}-M_{di}^{*}\right|\;\;\;\;\;\;\;NP^{\left(2\right)}=\frac{1}{N}\left[\sum_{j=1}^{n}\left|r_{ij}^{*}-M_{di}^{*}\right|/M_{di}\right]\;\;\;\\ {\mathrm{NP}}^{\left(3\right)}=\frac{\sqrt{\frac{\sum (r_{ij}^{*}-{\bar{r}}_{i.}^{*}{)}^{2}}{N}}}{{\bar{r}}_{i.}}\;\;\;\;\;\;{\mathrm{NP}}^{\left(4\right)}=\frac{2}{N\left(N-1\right)}\left[\sum_{j=1}^{n-1}\sum_{\left[j^{\prime }=j+1\right]}^{n}\left|r_{ij}^{*}-r_{ij^{\prime }}^{*}\right|/{\bar{r}}_{i.}\right] \end{array}$$

In the above relations $r_{ij}^{*}$ is the rank of *i*th genotype in the *j*th environment based on adjusted data, ${\bar{r}}_{ij}^{*}$ is the mean ranks for adjusted data, $M_{di}^{*}$ the median ranks for adjusted data, and ${\bar{r}}_{i.}$ and Mdi are the same parameters obtained from the original data (unadjusted). Low values of these statistics indicate high stability.

In general, non-parametric stability parameters are easy to use and interpret. Furthermore, any deletions or additions of one or a few genotypes have no significant effects on the results. In this regard, if breeders are only interested in the existence of rank order differences across environments, these methods provide the best alternative to parametric models currently used. Hence, the relative comparisons of the tested genotypes are more important than absolute comparisons. As non-parametric methods are based on rank values, a target genotype is considered stable if it reveals a constant ranking pattern across different environments. There are numerous reports related to the use of non-parametric methods in analyzing the GEI effect and selection of stable genotypes in different crops. For instance, Ahmadi et al. [46] used a set of non-parametric methods to investigate stability of grain and forage yields in 14 advanced lines of grass pea in semi-warm regions of Iran for three consecutive years. According to their results, among non-parametric parameters, the TOP parameter showed a dynamic concept of stability and showed a strong correlation with grain and forage yields. In a study conducted by Karimizadeh et al. [47], non-parametric statistic S^(6)^ showed a dynamic concept of stability and well-recognized high-yielding and stable lentil genotypes in a MET experiment. Khalili and Pour-Aboughadareh [48] evaluated yield stability and adaptability of 40 barley doubled haploid lines in eight environments, reporting a dynamic concept of stability for TOP parameter. However, Sabaghnia et al. [49] reported a positive correlation between grain yield and NP^(2)^, NP^(3)^, and NP^(4)^ stability parameters in durum wheat genotypes, and also showed a dynamic concept of stability for these measurements. Alizadeh et al. [50] investigated the GEI effect in a set of winter rapeseed lines, demonstrating that parameters such as S^(2)^, S^(3)^, S^(6)^, NP^(2)^, NP^(3)^, and NP^(6)^ along with KR, due to their strong correlation with seed yield, which enabled them to identify high-yielding and stable lines. Afzl et al. [51] reported that among non-parametric stability statistics, S^(1)^, S^(3)^, S^(6^,^)^ and NP^(4)^ with a dynamic concept are superior to evaluate high-yielding and stable safflower genotypes. In another study in canola, Mortazavian and Azizinia [52] reported that for nonparametric methods, TOP, *Si^(1)^*, and *RK* parameters were useful in detecting the stability of the genotypes. Non-parametric analysis of the phenotypic stability in chickpea genotypes showed that TOP and *RK* parameters are ideal measurements for identify the most stable genotypes [53]. Furthermore, Vaezi et al. [54] have highlighted the usefulness of RK, S^(3)^, S^(6)^, NP^(2)^, NP^(3)^, and NP^(4)^ parameters in selecting high-yielding and stable barley genotypes across different diverse environments.

### 3.2. Parametric Stability Statistics

#### 3.2.1. Environmental Variance (S^2^)

Romer [16] proposed the variance of yield performance for test genotypes across environments as a stability parameter. The mathematical equation for this parameter is as follows:

$$S_{}^{2}=\sum {\left(R_{ij}-m_{i}\right)}^{2}/(e-1)$$

where R_ij_ is yield of *i*th genotype in the *j*th environment, mi is grand mean yield across all environments, and e is the number of environments. The minimum value of S^2^ refers to the greatest stability. Derived stability measures include the square root value (S) and its coefficient of variation.

#### 3.2.2. Mean Variance Component (θ)

Plaisted and Peterson [17] proposed the variance component of GEI for interactions between each of the possible pairs of genotypes. This statistic considers the average of the estimate for all combinations with a common genotype to be a measure of stability. This stability statistic is described by the following equation:

$$\theta_{}=\frac{p}{2(p-1)(q-1)}\sum_{j=1}^{q}{\left(x_{ij}-{\bar{x}}_{i.}-{\bar{x}}_{.j}+{\bar{x}}_{..}\right)}^{2}+\frac{SSGE}{2(p-2)(q-1)}$$

$$SSGE=\sum \sum (x_{ij}-{\bar{x}}_{i.}-{\bar{x}}_{.j}+{\bar{x}}_{..}{)}^{2}$$

In the above equation, X_ij_ is the grain yield of genotype *i*th in environment *j*th; ${\bar{X}}_{i.}$ is the mean grain yield of genotype *i*th; ${\bar{X}}_{.j}$ is the mean grain yield of the environment *j*th; ${\bar{X}}_{..}$ is the grand mean; SSGE is the GEI sum square; and p and q are the numbers of genotypes and environments, respectively. Based on this statistic, the genotypes that show a lower value for 𝜃𝑖 are considered more stable.

#### 3.2.3. GE Variance Component (θ′)

This statistic is a modified measure of stability parameter. As shown in the following equation, *i*th genotype is deleted from the entire set of data, and the GEI variance from this subset is the stability index for the *i*th genotype [18].

$${\theta^{\prime }}_{}=\frac{-p}{\left(p-1)(p-2)(q-1\right)}\sum_{j=1}^{q}{\left(x_{ij}-{\bar{x}}_{i.}-{\bar{x}}_{.j}+{\bar{x}}_{..}\right)}^{2}+\frac{SSGE}{\left(p-2)(q-1\right)}$$

where X_ij_ is the grain yield of genotype *i*th in environment *j*th; ${\bar{X}}_{i.}$ is the mean grain yield of genotype *i*th; ${\bar{X}}_{.j}$ is the mean grain yield of the environment *j*th; ${\bar{X}}_{..}$ is the grand mean; SSGE is GEI sum square; and *p* and q are the numbers of genotypes and environments, respectively. Based on this statistic, the genotypes that show higher values for this statistic are considered more stable.

#### 3.2.4. Wricke’s Ecovalence ($W_{}^{2}$)

Wricke [19] proposed the concept of ecovalence as the contribution of each genotype to the GEI sum of squares. The ecovalence (W) of the *i*th genotype is its interaction with the environments, squared and summed across environments. Thus, genotypes with low values have smaller deviations from the mean across environments and are more stable. The following equation shows the mathematical process of this stability statistic:

$$W_{}^{2}=\sum {{(X}_{\mathrm{ij}}-{\bar{X}}_{i.}-{\bar{X}}_{.j}+{\bar{X}}_{..})}^{2}\;$$

where X_ij_ is the grain yield of genotype *i*th in environment *j*th; ${\bar{X}}_{i.}$ is the mean grain yield of genotype *i*th; ${\bar{X}}_{.j}$ is the mean grain yield of the environment *j*th; and ${\bar{X}}_{..}$ is the grand mean.

#### 3.2.5. Joint Regression Analysis (JRA)

The joint regression (JR) model was first suggested by Yates and Cochran [55]. This model was proposed again by Finlay and Wilkinson [20], Eberhart and Russell [21], and Perkins and Jinks [56], with slight variations, for use in stability analysis and to identify the stable genotypes in various environments. The JR model is as follows:

$$Y_{ij}=\mu +G_{i}+E_{j}+b_{i}E_{j}+d_{ij}+e_{ij}$$

In the above equation, Y_ij_, μ, G_i_, E_j_, b_i_, d_ij_, and e_ij_ are the mean yield for *i*th genotype in *j*th environment, the mean of all genotypes, the effect of genotype *i*, the effect of environment *j*, the linear coefficient of the *i*th genotype on environmental index, deviation from regression, and the average of the random errors associated with genotypes and environments, respectively.

In the regression model, the GEI is explained in terms of differential sensitivities to the improvement of the environment, with some genotypes benefiting more than others from an increase in environmental quality. Furthermore, the regression model has the advantage that researchers can use unbalanced data in a univariate model, whereas the other methods need to be balanced. Finlay and Wilkinson [20] believed that the b_i_ statistic can measure the stability and relative adaptability, while Eberhart and Russell [21] developed this concept by computing the deviations from linear regression ($S_{di}^{2}$) statistic. Thus, the regression coefficient (b_i_) and variance deviation ($S_{di}^{2}$) are the two main components of the RJ model. Accordingly, genotypes with b = 1 and $S_{di}^{2}$ = 0 (minimum value) are highly stable. When this feature is associated with high mean performance, genotypes show general adaptability, and in contrast, when associated with low mean yield, genotypes indicate poor adaptation to all environments. The bi values greater and lower of 1 explain other important concepts; a b_i_ > 1 shows genotypes that are responsive to high yielding environments, while a b_i_ < 1 indicates genotypes that are responsive to low-yielding environments.

#### 3.2.6. Tai’s Stability Statistics

In this model of stability, the GEI is partitioned into two components as described by Tai [22]: (1) the linear response to environmental effects, which is estimated by the α statistic, and (2) the deviation from the linear response, which is calculated by the λ statistic. According to this model, genotypes with α = −1 and λ = 1 have the highest stability, whereas genotypes with α = 0 and λ = 1 show an average stability across environments. This model also provides a graphical tool for the prediction interval for α = 0 and a confidence interval for λ values; in this way, the test genotypes can be dispersed in different stability regions of Tai’s plot.

#### 3.2.7. Shukla’s Stability Variance (σ^2^)

Shukla [23] suggested the stability variance of genotype *i*th as its variance across environments after the main effects of environmental means have been removed. According to this statistic, genotypes with minimum values are more stable. This statistic is calculated based on the following equation:

$$\;\sigma_{}^{2}=\left[\frac{p}{\left(p-2)(q-1\right)}\right]\;\;W_{}^{2}\;-\frac{\sum W_{i}^{2}}{\left(p-1)(p-2)(q-1\right)}\;$$

where W^2^ is Wricke’s ecovalence, and p and q are the numbers of genotypes and environments, respectively.

#### 3.2.8. Pinthus’s Coefficient of Determination (R^2^)

This statistic is defined as predictability of response suggested by Pinthus [24] as another stability parameter, in which a variation of mean yield was explained by genotype response across environments. This parametric statistic can be described with the following equation:

$$R_{}^{2}=\frac{b_{i}^{2}\sum {\left({\bar{x}}_{.j}-\bar{x}..\right)}^{2}}{\sum {\left(x_{ij}-{\bar{x}}_{i.}\right)}^{2}}$$

where b_i_ is slope regression, X_ij_ is the grain yield of genotype *i*th in environment *j*th; ${\bar{X}}_{i.}$ is the mean grain yield of genotype *i*th; ${\bar{X}}_{.j}$ is the mean grain yield of the environment *j*th; and ${\bar{X}}_{..}$ is the grand mean. A genotype with the highest value is intended to be more stable.

#### 3.2.9. Coefficient of Variance (CV)

The coefficient of variation is suggested by Francis and Kannenberg [25] as a parametric stability statistic through the combination of the coefficient of variation, mean yield, and environmental variance.

$$CV_{}=\frac{SD_{x}}{\bar{X}}\times 100$$

where SD_x_ is the standard deviation of a genotype mean across environments and is the grand mean. Genotypes with low CV, low environmental variance, and high mean yield are considered the most desirable. Furthermore, by plotting the mean yield (x axis) against CV values (y axis), test genotypes can be divided into four groups: Group I including genotypes with high yield and small variation; Group II including genotypes with high yield and large variation; Group III including genotypes with low yield and small variation; and Group IV including genotype with low yield and large variation.

#### 3.2.10. Superiority Index (P)

The mean square of distance between the genotype’s response and the maximum response over environments is defined as superiority index (P) [28]. A low value of Pi indicates high relative stability. Furthermore, the following equation shows mathematical relations for this statistic:

$$P=\sum_{j=1}^{n}{\left(X_{ij}-M_{j}\right)}^{2}/(2n)$$

where n is the number of environments, X_ij_ is the yield of the *i*th genotype in the *j*th environment, and M_j_ is the maximum response (yield) among all genotypes in the *j*th environment.

#### 3.2.11. AMMI-Based Stability Statistics

A complete description of GEI requires more sophisticated models or approaches than the analysis of variance (ANOVA). The ANOVA is an additive model that only explains main effects and determines if GEI is a significant source of variation, but it cannot provide further information to highlight the patterns of genotypes (G) and or environments (E) that give rise to the GEI. Principal component analysis (PCA), as a multivariate technique, is a useful model that includes no sources of variation for additive main effects of G or E and does not analyze the interactions effectively. The additive main effects and multiplicative interaction analysis, which is identified as an AMMI model, consists in fitting an additive model (ANOVA) for general means, G’s and E’s means, and multiplicative model (PCA) for the residual of an additive model or a GEI. Hence, AMMI is a better model for analysis of the GEI in a MET, because it not only provides an estimate of the total GEI effect of each genotype but also partitions it into several interaction effects due to individual environments. Furthermore, the AMMI model provides an easy interpretation of the obtained results by the graphical biplot tool to stratify genotypes and the environment [57]. In general, this model can be used as an effective analytical approach in terms of several aspects: (i) understanding GEI, (ii) improving the accuracy of yield estimates, (iii) identifying mega-environment patterns, (iv) increasing the flexibility of experimental designs, and (v) imputing missing data [58,59]. Zobel et al. [57] combined the standard ANOVA with PCA analysis and proposed the following equation for AMMI model:

$$Y_{ij}=\mu +g_{i}+e_{j}+\sum_{n=1}^{N}\lambda_{n}\gamma_{in}\delta_{jn}+\rho_{ij}+\varepsilon_{ij}$$

where μ, g_i_, and e_i_ are the grand mean, the main effect of the genotype *i*, and the main effect of environment *j*, respectively. The GEI will explain by, where λ_n_, γ_in_, and δ_jn_ are the eigenvalue of the nth interaction PCA (IPCA) retained in the AMMI model, the eigenvector for the *i*th genotype from nth IPCA, and the eigenvector for the *j*th environment from the nth IPCA, respectively. N, ρ_ij_, and ε_ij_ indicate the number of IPCA retained in the AMMI model, the GEI residual, and the random error, respectively. Based on the AMMI’s output, several stability statistics have been proposed by different researchers for evaluating genotypes. The following equations describe these statistics.

##### Averages of the Squared Eigenvector Values (EV)

The average of the squared eigenvector value (EV) parameter was proposed by Zobel [32]. According to the following equation, three parameters EV_1_, EV_V_, and EV_F_ are computed as follows:

$${\mathrm{EV}}_{i}=\sum_{n=1}^{N}\frac{\gamma_{in}^{2}}{N}$$

For EV_1_, N is one; for EV_V_, N is the number of IPC that retain in the AMMI model via validation procedures; and for EV_F_, N is the number of IPC that retain the AMMI model via *F*-test. Genotypes with the lowest values for these statistics are identified as the most stable.

##### Sums of the Absolute Value of the IPC Scores (SIPC)

Based on the AMMI’s results, three other stability statistics were suggested by Sneller et al. [34]. These statistics (SIPC_1_, SIPC_V_, and SIPC_F_) are calculated based on sums of the absolute value of the IPC scores for each test genotype according to the following equation:

$$\mathrm{SIPC}=\sum_{n=1}^{N}\left|\lambda_{n}^{0.5}\gamma_{in}\right|$$

Similar to the EV statistic, for SIPC_1_, N is one; for SIPC_V_, N is the number of IPCs that are retained in the AMMI model via validation procedures; for SIPC_F_, N is the number of IPCs that retain in the AMMI model via *F*-tests. λ_n_ and γ_in_ are the eigen value of the nth IPCA that is retained in the AMMI model and the eigenvector for the *i*th genotype from the nth IPCA, respectively. The lowest values for these statistics showed the highest stability.

##### Sum across Environments of the GEI Modeled by AMMI (AMGE)

The further three AMMI-based stabilities are the sum across environments of the GEI modeled by AMMI [34]. Similar to SIPCs, there are three forms of this statistics:

$${\mathrm{AMGE}}_{}=\sum_{N}^{M}\sum_{n=1}^{N}\lambda_{n}\gamma_{in}\delta_{jn}$$

where λ_n_, γ_in_, and δ_jn_ are the eigenvalue of the *n*th interaction PCA (IPCA) retained in the AMMI model, the eigenvector for the *i*th genotype from nth IPCA, and the eigenvector for the *j*th environment from the nth IPCA, respectively. For AMGE_1_, N is one; for AMGE_V_, N is the number of IPCs that are retained in the AMMI model via validation procedures; for AMGE_F_, N is the number of IPC that retain in the AMMI model via *F*-tests. Lower values of these statistics showed the highest stability.

##### Distance of IPCAs Point from Origin in Space (D)

Annicchiarico [35] proposed two AMMI-based statistics through the distance of IPCA point with origin in space (D). In other words, these statistics provide the GEI estimate of a particular genotype with a group of environment samples. In this way, the greater the D value of a genotype, the greater the distance of the genotype from the origin of IPCAs. Hence, the genotype with the lowest value of these statistics would be the most stable. The following equation provides the mathematical formula for D:

$$D_{a}={\left[\sum_{n=1}^{N}{\left(\lambda_{n}\gamma_{in}\right)}^{2}\right]}^{0.5}$$

where λ_n_ and γ_in_ are the eigenvalue of the *n*th IPCA retained in the AMMI model and the eigenvector for the *i*th genotype from nth IPCA, respectively.

Zhang et al. [36] proposed another form of this statistic as D_Z_:

$$D_{z}={\left[\sum_{n=1}^{N}{\left(\gamma_{in}\right)}^{2}\right]}^{0.5}$$

Similarly to D_a_, genotypes with the lowest value of this statistic are the most stable.

##### AMMI Stability Value (ASV)

Purchase et al. [37] developed another stability statistic based on the two first IPCA scores for each genotype. The AMMI stability value (ASV) is the distance from the coordinate point to the origin in a two dimensional scattergram of IPCA1 scores against IPCA2 scores. This measurement is described as follows:

$${\mathrm{ASV}}_{}=\sqrt{{\left(\frac{SS_{IPCA1}}{SS_{IPCA2}}(IPCA1)\right)}^{2}+{\left(IPCA2\right)}^{2}}$$

where SSIPCA1/SSIPCA2 is the ratio between the sum of squares from the first and second interaction principal component axis, and IPCA1 and IPCA2 are the genotypic scores of these components in the AMMI model. The genotype with the lowest value of this statistic would be more stable.

##### Stability Measure Based on Fitted AMMI Model (W_(AMMI)_)

Raju [38] proposed a measure of stability that may be viewed as Wricke’s ecovalance (W^2^). Because this statistic is calculated based on the AMMI model, it is denoted by W_(AMMI)_.

$$W_{\left(\mathrm{AMMI}\right)}=\sum_{n=1}^{N}\lambda_{n}^{2}\gamma_{ni}^{2}$$

In the above equation, λ_m_ and γ_in_ are the singular value for the PCA axis and the *i*th genotype IPCA score for the axis, respectively. N is the number of significant IPCAs. Therefore, it can be stated that the stability rank order obtained from W_(AMMI)_ is equivalent to that of W^2^. The genotype with the lowest value of this statistic would be the most stable. It is worth noting that when the first IPCA only is retained in the AMMI model, this statistic can be changed to the FP statistic:

$${\mathrm{FP}}_{}=\lambda_{l}^{2}\gamma_{1i}^{2}$$

In this situation, $\lambda_{1}^{2}$ is same for all genotypes; thus, the absolute value γ is sufficient for comparison, and a lower value of γ explains the greater stability. The comparison of genotypes for stability based on this statistic will be equivalent to the comparison based on the biplot with first IPCAs axis. If the two first IPCAs are retained in the AMMI model, stability comparisons will be equivalent to the comparisons based on biplots with the first two PCA axes. Indeed, its equation will be as follows:

$$B=\sum_{n=1}^{2}\lambda_{n}^{2}\gamma_{ni}^{2}$$

This is clear that the last statistics will be less precise than W_(AMMI)_, as is evident from the fact that they could not exploit the information in detail. In other words, the reliability of a stability statistic improves with the increase in the number of axes retained in the model.

##### AMMI Based Stability Parameter (ASTAB)

The AMMI-based stability parameter (ASTAB) was proposed by Rao and Prabhakaran [39] as follows:

$$ASTAB=\sum_{n=1}^{N}\lambda_{n}^{}\gamma_{in}^{2}$$

where λ_n_ and γ_in_ are the eigenvalue of the nth IPCA and the eigenvector value for the *i*th genotype. A genotype is considered to be more stable when the value of this statistic is lower.

##### Genotype Stability Index (GSI)

Farshadfar [41] developed a stability statistic based on the rank of mean yield of genotype across environments (RY) and rank of ASV value (RASV). According to this criterion, a genotype with the highest value of GSI would be more stable.

$$GSI=RASV_{}+RY_{}$$

##### Modified AMMI Stability Value (MASV)

The modified AMMI stability values (MASV) was suggested as another stability statistic by Zali et al. [42] as follows:

$${\mathrm{MASV}}_{}=\sqrt{\sum_{n=1}^{N-2}{\left(\left(\frac{SSIPCA_{n}}{SSIPCA_{n+1}})(IPCA_{n}\right)\right)}^{2}+{\left(IPCA_{N}\right)}^{2}}$$

The main difference between MASV and ASV is the use of all significant IPCAs in the MASV statistic. Similar to ASV, a genotype with the lowest value of MASV will be selected as the most stable.

##### Absolute Value of Relative Contribution of IPCAs (Za)

Zali et al. [42] also proposed the absolute value of relative contribution IPCA as another statistic to measure of stability.

$${\mathrm{Za}}_{}=\sum_{i=1}^{N}\left|\theta_{n}\gamma_{in}\right|$$

where θ_n_ is the percentage sum of squares explained by the nth IPCA and N is the number of IPC that are retained in the AMMI model via the *F*-test. Lower values of *Za* show the highest stability.

##### Sum across Environments of Absolute Value of GEI Modeled by AMMI (AV(AMGE))

The third stability statistic developed by Zali et al. [42] is the sum across environments of the absolute value of genotype × environment interaction modeled by AMMI (AV_(AMGE)_). This statistic is calculated as follows:

$${\mathrm{AV}}_{(\mathrm{AMGE}}{}_{)}=\sum_{j=1}^{E}\sum_{n=1}^{N}\left|\lambda_{n}\gamma_{in}\delta_{jn}\right|$$

where λ_n_, γ_in_, and δ_jn_ are the eigenvalue of the nth interaction PCA (IPCA) retained in the AMMI model, the eigenvector for the *i*th genotype from nth IPCA, and the eigenvector for the *j*th environment from the nth IPCA, respectively. N is the number of significant IPCs retained in the AMMI model via *F*-test. A genotype with the lowest value of AV_(AMGE)_ will be selected as the most stable

##### AMMI Stability Index (ASI)

The AMMI stability index (ASI) is another methodology for measuring stability proposed by Jambhulkar et al. [43] as follows:

$${\mathrm{ASI}}_{}=\sqrt{\left[{\left(\mathrm{IPCA}1\times \theta_{1}^{2}\right)}^{2}+{\left(IPCA2\times \theta_{2}^{2}\right)}^{2}\right]}$$

where IPCA1 and IPCA2 are the scores of the two first principal component interactions, respectively. $\theta_{1}^{2}$ and $\theta_{2}^{2}$ are percentage sum of squares explained by the first two IPCAs effects, respectively. The lowest value of this statistic shows the most stability.

##### Modified AMMI Stability Index (MASI)

Ajay et al. [44] modified the ASI statistic as follows:

$$\mathrm{MASI}=\sqrt{\sum_{n=1}^{N}{\mathrm{IPCA}}_{n}^{2}\times \theta_{n}^{2}}$$

Unlike ASI, MASI computes stability value considering all significant IPCAs in the AMMI model. In the above equation, IPCAn and $\theta_{n}^{2}$ are the scores of the *n*th IPCA and the percentage sum of square explained by the nth IPCAs effects, respectively. Similar to ASI, the lowest values show the most stability.

#### 3.2.12. BLUP-Based Stability Statistics

The best linear unbiased prediction (BLUP) is known as the best methodology for the estimation of random effects in the linear model [60]. Using the BLUP and the restricted maximum likelihood (REML), several parameters were proposed for measuring performance and stability simultaneously [40]. The first parameter is the harmonic mean of genotypic values (HMGV) that identifies the genotype with the highest harmonic mean across environments, as the most stable, as follows:

$${\mathrm{HMGV}}_{}=\frac{E}{\sum_{j=1}^{E}\frac{1}{GV_{ij}}}$$

The second parameter is the relative performance of genotypic values (RPGV), which is considered as an adaptability index and computed as follows:

$${\mathrm{RPGV}}_{}=\frac{1}{E}\sum_{j=1}^{E}GV_{ij}/\mu_{j}$$

The harmonic mean of RPGV (HMRPGV) is the third BLUP-based stability parameter that considers stability, adaptability, and mean performance simultaneously. This parameter is calculated as follows:

$${\mathrm{HMRPGV}}_{}=\frac{E}{\sum_{j=1}^{E}\frac{1}{GV_{ij}/\mu_{j}}}$$

In the above formulas, GV_ij_, μ_j_, and E are the genotypic values (BLUP) for the *i*th genotype in the *j*th environment, the grand mean for each environment j, and the number of environments, respectively. As has been mentioned by Resende et al. [40], the highest values of these parameters are suitable.

#### 3.2.13. The Weighted Average of Absolute Scores (WAASB)

Recently, Olivoto et al. [45] developed an interesting integrated stability statistic based on AMMI and BLUP models. This statistic is the weighted average of absolute scores from the singular value decomposition of the matrix of the best linear unbiased predictions for the genotype × environment interaction effects generated by a linear mixed-effect model. This index is estimated as follows:

$${\mathrm{WAASB}}_{}=\frac{\sum_{k=1}^{p}\left|IPCA_{ik}\times EP_{k}\right|}{\sum_{k=1}^{p}EP_{k}}$$

where IPCA_ik_ is the score of the *i*th genotype (or environment) in the *k*th IPCA, and EP_k_ is the amount of the variance explained by the *k*th IPCA. According to this statistic, a genotype with the lowest WAASB value is considered the most stable. Furthermore, for identifying highly productive and stable genotypes, we can also use a biplot based on the WAASB and grain yield. Indeed, in this way, all the estimated IPCA axes contribute to identifying the stability in a bi-dimensional plot.

Thus far, many research articles and notes have been released regarding the importance of the GEI effects and various parametric statistical models (e.g., Fasahat et al. [61]; van Eeuwijk et al. [62]; Malosetti et al. [63]); however, based on the nature of data in each trial, researchers use some of the stability approaches and models to identify stable genotypes and target test environments. On the other hand, there are numerous reports available regarding the applicability of these methods to select stable varieties in METs (see van Eeuwijk et al. [62]); here, we highlighted some of key studies to show the importance of them in the breeding programs. For instance, Zali et al. [42] used a set of AMMI-based stability statistics to identify of most stable chickpea genotypes. In a study conducted by Dehghani et al. [64], integrating parametric and nonparametric statistics results in identifying the most stable tall fescue genotypes in METs. Additionally, these authors declared that the GSI index with a dynamic concept of stability is an ideal parameter to select superior genotypes. Burbano-Erazo et al. [65] used a set of AMMI-based methods to test the stability of common bean genotypes for heat and drought environments. They reported that the ASV and YSI parameters allow selecting stable genotypes across environments. Integrating some parametric and non-parametric stability indices in a study conducted by Vaezi et al. [2] showed that the bi and CV parameters have a positive and significant correlation with grain yield in a set of barley genotypes. Indeed, these parameters provided a measure of stability in a dynamic sense.

Ajay et al. [66] exploited 12 AMMI-based stability parameters and simultaneous selection for yield and stability (SSI) to select superior genotypes of peanut when 52 genotypes were tested for two years under two phosphorus levels. In this experiment, AMMI-based stability parameters such as ASI, ASV, ASTAB, AVAMGE, Da, EV, FA, MASI, and SIPC showed a positive and significant correlation with mean yield. Moreover, they stated that although the mentioned parameters have a dynamic concept of stability, only some of them, such as SIPC, MASI, and MASV, were useful for the identification of stable high-yielding genotypes. Verma and Singh [67] showed that among a collection of parametric AMMI- and BLUP-based stability parameters, the superiority indices would provide reliable estimates of genotype performance. Among parametric methods, YSI and HMRPGV simultaneously provide a status of stability and productivity. HMRPGV measures genotypic stability and productivity in METs. Indeed, this parameter provides simultaneous selection for productivity and phenotypic stability in the context of a mixed model. Results of the study performed by Mahadevaiah et al. [68] in the delineation of genotype × environment interaction for identification of stable genotypes of sugarcane revealed that screening of drought-tolerant and stable genotypes using the GSI parameter has a considerable association with the multi-environment BLUP results, so this parameter could be used as an ideal indicator in the similar experiments. Verma and Singh [67] identified the high-yielding and stable genotypes of wheat using HMRPGV parameter. In regard to YSI parameter, Jamshimoghaadm and Pourdad [69], in a study of the effects of GEI on seed yield in spring safflower genotypes, reported a dynamic concept of stability for it and stated that this parameter is a useful tool for identifying ideal genotypes in MET experiments. Agyeman et al. [70] used the REML and BLUP model to test the stability of a set of maize genotypes in a MET trial. Furthermore, they were able to predict the genetic gain and accrue estimations of performance using the BLUP model. In a comparative study, Anuradha et al. [71] used a set of AMMI- and BLUP-based stability statistics to select stable and high-yielding finger millet genotypes. Based on the obtained results, they stated that several statistics, such as ASV, ASTAB, AVAMGE, D_A_, Dz, EV, and FA, proved that all have equal potential in the identification of stable genotypes.

Stability analyses are evolving continuously to add precision to the GEI component. Instead of depending upon a single method and approach, papers published analyzing this effect and the prediction of phenotypic stability of various crops are often found in the integration of several models. The main objective in each study is to select the correct method or model of analysis to capture the maximum GEI effect. Although the AMMI and BLUP models are the most commonly used models so far, each of them has some disadvantages. To solve this problem, Olivoto et al. [45] combined the features of both models and developed a unique stability parameter and named it WAASB. Indeed, this model is the newest model, and so far, its merit has been continuously evaluated by breeders. In barley, Pour-Aboughadareh et al. [72,73] exploited the WAASB parameter to identify the high-yielding and stable genotypes of barley. They reported that this parameter can be ideal index for identify superior varieties in METs experiments. Koundinya et al. [74] used the WAASB parameter to select of stable cassava genotypes under drought environments. Nataraj et al. [75] evaluated the usefulness of the WAASB parameter to identify the high-yielding and stable genotypes of soybean in a METs experiment and reported a good capability of this model in the grouping of the genotypes based on their performance and stability.

#### 3.2.14. GGE Biplot Approach

The GGE biplot methodology is a graphical tool that superbly helps breeders to interpret the GEI in MET experiments. The first theory of this methodology was described by Yan [76], and then numerous studies used this method and reported its advantage compared with other numerical methods. The GGE biplot includes of a set of biplot interpretation models, whereby important questions regarding genotype and environment evaluation can be visually addressed [77]. The detailed description of this methodology and interpretation of each biplot can be found in the review of Yan and Tinker [77]. In this section we address how this methodology could help the breeder to interpret the GEI effect, and to select the ideal genotypes across different environments with high yield and the most stability.

The most important application of this methodology can summarized as follows [75]:

(1) Providing a ranking pattern for test genotypes based on their yield performance of any specified environment;
(2) Providing a ranking pattern for test environments based on the relative yield performance of any specified genotype;
(3) Comparing the yield performance of any given pair genotypes across environments
(4) Recognizing the best genotype(s) in each test environment;
(5) Identifying potential mega-environments based on the best genotype;
(6) Simultaneously investigating the genotypes based on stability and average performance;
(7) Determining discriminating ability and representativeness power of test environments;
(8) Visualizing all the above features for a subset of the data by removing some of the genotypes or environments.

Two stability indices, the GGE Distance (GGED) and GGE Instability index (GGEIN), can be calculated by the GGE biplot model. The GGED measures the distance of each genotype from the ‘‘ideal’’ genotype, which is defined as the virtual genotype that has the highest mean performance and stability. The GGEIN parameter approximates the genotype’s contribution to the GEI [78]. The GGE biplot methodology is one of the analytical tools, which alone or in combination with other methods is commonly used for the analysis of the GEI in various crops. There are numerous reports in the literature on the applicability of this methodology in breeding programs. Hence, to show the importance of this graphical tool in various breeding programs, we have only highlighted some of the papers published during the last two years (2020–2022) for each crop (Table 2).

**Table 2:** Some examples of the use of the GGE biplot methodology in METs in different crops.

Crop	Number of Genotypes	Number of Environments	Target Trait	References
Mung bean	22	12	Resistance to leaf spot	[79]
Pyrethrum	10	4	Dry flower yield	[80]
Sorghum	324	3	Grain yield/Panicle weight	[81]
Sorghum	22	24	Grain yield	[82]
Groundnut	95	4	Grain yield	[83]
Potato	50	3	Tuber yield	[84]
Soybean	6	16	Grain yield	[85]
Cowpea	27	18	Grain yield	[86]
Chickpea	126	24	Resistance to *Fusarium*	[87]
Barley	20	12	Grain yield	[88]
Sunflower	11	16	Grain yield	[89]
Sugarcane	16	4	Cane yield	[90]
Common vetch	6	8	Forage yield	[91]
Rice	103	6	Grain yield	[92]
Melon	36	3	Fruit yield	[93]
Wheat	24	24	Grain yield	[94]
Maize	15	7	Grain yield	[95]
Pigeonpea	15	5	Grain yield	[96]
Cotton	21	8	Seed yield	[97]
Durum wheat	5	16	Seed quality	[98]

## 4. How We Can Compute Stability Statistics?

Progress in computer sciences and programming languages has resulted in the advent of various script codes and software that help breeders to analyze the big data sets in their experiments. In other words, the breeder using these tools can better interpret the GEI effect and select the best genotypes with an acceptable accuracy. In this section, we have tried to address any macro codes, script, and software that enable computation of stability statistics., Table 3, Table 4 and Table 5 provide more information regarding features and capability of each software and packages. The features of each software are shown in Table 3.

**Table 3:** Features of existing software for analyzing the GEI effect in METs experiments.

Feature	GGE	GENES	GenStat	IRRISTAT	AMMISOFT	GEA-R	STABILITYSOFT
Windows support	√	√	√	√	√	√	√
Unix/Linux support			√				√
Mac OSX support			√				√
Portable							√
GUI (graphical user interface)	√	√	√	√	√	√	√
Offline usage capability	√	√	√	√	√	√	√

### 4.1. GGE Biplot Software

This software specialist computes the GGE biplot analysis. The results obtained by this software will be graphically released [76].

### 4.2. GENES

GENES is a software package used for data analysis and processing with different biometric models for genetic studies applied to plant and animal breeding. It allows parametric and non-parametric stability statistics to be computed and presents integration with MS Word, MS Excel and Paint. It is also compatible with the free software R and Matlab, through the supply of useful scripts available for complementary analyses in different areas [99].

### 4.3. GenStat Software

This software is a Windows-based statistical tool [100]. Using this software, the researcher can compute the AMM model and GGE biplot analyses.

### 4.4. AMMISOFT

Gauch and Moran [101] developed a Windows-based software that enables breeders to understand complex GEI and AMMI models. Using this software, breeders can obtain more information about AMMI families.

### 4.5. GEA-R Software

Genotype × Environment Analysis with R for Windows is a user-friendly and free software developed in the International Maize and Wheat Improvement Center (CIMMYT) by Pacheco et al. [102]. This software can perform procedures analysis for the AMMI model, site regression GGE biplot (SREG), partial least squares (PLS), and factorial regression and computes several parametric and non-parametric stability statistics.

### 4.6. IRRISTAT Software

This software is a free and user-friendly tool that was developed by the Biometrics Unit at the International Rice Research Institute (IRRI) [103]. Using this software, the AMMI model and some stability statistics can be computed.

### 4.7. STABILITYSOFT Software

Pour-Aboughadareh et al. [104] developed another free and user-friendly web-based software to compute several parametric and non-parametric stability statistics. This software provides an R script code for all estimated statistics. This software is compatible with UNIX platforms, Windows, and MacOSX, and it provides information on both Pearson’s and Spearman’s rank-order correlation coefficients among measured stability statistics.

### 4.8. SAS

Over the past thirty years, various macro codes have been released by researchers to compute some stability statistics. Piepho [105] published the first SAS’s code, which only was able to calculate the Shukla’s stability variance ($\sigma_{}^{2}$), coefficient of variance (CV), and deviation from regression ($S_{di}^{2}$). After then, Hussein et al. [106], Akbarpour et al. [107], and Dia et al. [108] developed other codes that enable breeders to calculate other stability statistics. Among these, the SAS macro code developed by Dia et al. [108]––which has been called SASG×E––is more complete compared to other codes.

### 4.9. Scripts for R Software

R is identified as a free software environment for statistical computing and graphics. This software is compatible with a wide variety of Windows, MacOS, and UNIX platforms. Due to this advantage, many researchers and plant breeders have been encouraged to create scripts for computing stability statistics. Until now, there have many R scripts that each enable calculation of some stability parameters. However, Olivoto and Lucio [109] developed an R package “metan” for analyzing the GEI effect in plant breeding experiments. This package enables breeders to compute the AMMI model and GGE biplot, as well as estimating several parametric and non-parametric stability statistics.

**Table 4:** The capability of different software for computing the stability statistics.

Statistic	Symbol	GGE Biplot	GENES	GenStat	IRRISTAT	AMMISOFT	GEA-R	STABILITYSOFT
Mean variance component	θ							√
GE variance component	θ′							√
Wricke’s ecovalence	W^2^		√				√	√
Regression coefficient	b_i_		√		√		√	√
Deviation from regression	$S_{di}^{2}$		√		√		√	√
Environmental variance	S^2^		√					
Tai’s stability statistics	λ and α		√				√	
Shukla’s stability variance	$\sigma_{}^{2}$		√				√	√
Pinthus’s coefficient of determination	R^2^				√		√	
Coefficient of variance	CV						√	√
Nassar and Huhn’s and Huhn’s statistics	S^(1, 2, 3, 6)^						√	√
Superiority index	P		√				√	
Kang’s rank-sum	KR							√
TOP-Fox	TOP							
Yield stability index	YS							
Averages of the squared eigenvector values	Ev							
Thennarasu’s non-parametric statistics	NP^(1−4)^							√
Sums of the absolute value of the IPC scores	SIPC							
Sum across environments of the GEI modeled by AMMI	AMGE							
Distance of IPCAs point with origin in space	D							
AMMI stability value	ASV							
Stability measure based on fitted AMMI model	W_(AMMI)_							
AMMI Based Stability Parameter	ASTAB							
Harmonic mean of genotypic values	HMGV							
Relative performance of genotypic values	RPGV							
Harmonic mean of RPGV	HMRPGV							
Genotype stability index	GSI							
Modified AMMI stability value	MASV							
Absolute value of relative contribution of IPCAs	Za							
Sum across environments of absolute value of GEI modeled by AMMI	AV_(AMGE)_							
AMMI stability index	ASI							
Modified AMMI stability index	MASI							
Weighted average of absolute scores	WAASB							
AMMI model	†	√	√	√	√	√	√	
GGE	††	√	√	√	√	√	√	

† AMMI model and related biplots; †† the biplot obtained by interpreting GEI effect.

**Table 5:** The capability of SAS and R macro- and script codes in computing stability statistics.

Statistic	Macro Codes for SAS				Packages and Codes for R						
					Phenability	Stability	Agrostab	Stabilitysoft	PBTools	Ammistability	Metan
θ		√						√			
θ′		√						√			
W^2^		√		√		√		√			√
b_i_		√		√		√	√	√	√		√
$S_{di}^{2}$	√	√		√		√	√	√	√		√
S^2^							√				
λ and α							√				
$\sigma_{}^{2}$	√			√		√	√	√	√		√
R^2^											√
CV	√	√		√			√	√			√
S^(1,2,3,6)^		√	√		√		√	√			√
P				√			√				√
KR					√	√		√			
TOP					√	√					√
YS				√							
Ev										√	√
NP^(1−4)^			√		√			√			√
SIPC										√	√
AMGE										√	√
D						√				√	√
ASV										√	√
W_i(AMMI)_										√	√
ASTAB										√	√
HMGV											√
RPGV											√
HMRPGV											√
GSI										√	√
MASV						√				√	√
Za										√	√
AV_(AMGE)_										√	√
ASI										√	√
MASI										√	√
WAASB											√
GGE				√		√					√
AMMI				√		√				√	√
References	[105]	[106]	[107]	[108]	[110]	[111]	[112]	[104]	[113]	[114]	[109]

## 5. Conclusions

It is clear that the selection of genotypes for target environment(s) is affected by the GEI effect. For this reason, over the three past decades, numerous statistical models and approaches have been proposed to analyze GEI as well as identify the high-yielding and most stable genotypes. This fact is not unexpected in that each stability parameter or statistic result shows a specialist ranking pattern for genotypes. Hence, in each experiment, it is best that plant breeders compute all statistics and ultimately select the superior genotypes based on their yield performance and stability, as it is clear there are several stability models and approaches to the analysis of the GEI effects in METs. Some of these models are based on the genotypic contribution to GE variance, and some are based on G + GE (e.g., univariate or multivariate). Models and/or methods based on G + GE are more repeatable if they are calculated within mega-environments because mean yield is more repeatable. It seems that to allow better decisions on the selection of the superior genotypes, a complex of parametric and non-parametric stability statistics can be used as an additional tool. Another issue we should bear in mind is the fact that GEI is always affected by biotic and abiotic factors, some of which are dynamic (e.g., insect–disease incidence), static (e.g., temperature, precipitation), or complex factors. Hence, in each experiment, attention to the causes of GEI is very important and should be dissected. In this review, we tried to gather all statistical models and their related statistics that can help breeders for their breeding aims. Furthermore, we anticipate that the presented information regarding software and packages can help breeders to accelerate their data analysis and to compare different models and methods.

## Data Availability

Not applicable.
